# Treatment of cricopharyngeal dysfunction: a comparative pilot study

**DOI:** 10.1186/s13104-015-1266-x

**Published:** 2015-07-10

**Authors:** Beatriz Arenaz Búa, Rolf Olsson, Ulla Westin, Roland Rydell, Olle Ekberg

**Affiliations:** Division of Logopedics, Phoniatrics and Audiology, Department of Clinical Sciences, Lund University, Skane University Hospital, Jan Waldenströmsgata 18, 205 02 Malmö, Sweden; Division of Ear, Nose and Throat Diseases, Head and Neck Surgery, Department of Clinical Sciences, Lund University, Skane University Hospital, Jan Waldenströmsgata 18, 205 02 Malmö, Sweden; Diagnostic Centre of Imaging and Functional Medicine, Department of Clinical Sciences, Lund University, Skåne University Hospital, 205 02 Malmö, Sweden; Division of Logopedics, Phoniatrics and Audiology, Department of Clinical Sciences, Lund University, 221 85 Lund, Sweden; Division of Ear, Nose and Throat Diseases, Head and Neck Surgery, Department of Clinical Sciences, Lund University, 221 85 Lund, Sweden; Diagnostic Centre of Imaging and Functional Medicine, Department of Clinical Sciences/Medical Radiology, Skåne University Hospital, Lund University, 205 02 Malmö, Sweden

**Keywords:** Cricopharyngeal dysfunction, Upper oesophageal sphincter, Cricopharyngeus muscle, Videomanometry, Sydney Swallowing Questionnaire

## Abstract

**Background:**

Cricopharyngeal dysfunction is a narrowing at the level of the upper oesophageal sphincter caused by failed or incomplete sphincter opening as a result of lack of pharyngoesophageal coordination or reduction in the muscular compliance of the upper oesophageal sphincter. Oropharyngeal dysphagia is a typical symptom. Videomanometry allows direct comparison of pressure readings with dynamic anatomy during swallowing.

**Methods:**

This is a prospective randomized pilot study that compares the effect of balloon dilatation and laser myotomy in cricopharyngeal dysfunction. We used videomanometry as an objective measure and the Swedish version of Sydney Swallowing Questionnaire as patient’s self-assessment at baseline and 1 and 6 months after treatment.

**Results:**

The UES sagittal diameter increased from 5.6 mm pre-operatively to 8.4 mm 6 months post-operatively with no differences between treatment groups. Preoperative mean Sydney Swallowing Questionnaire score was 770 and 6 months post-operative score 559, with no difference between the treatments in our cohort.

**Conclusion:**

Cricopharyngeal dysfunction treatment by either laser myotomy or balloon dilatation improved upper oesophageal sphincter opening during at least 6 months.

Trial registration: ISRCTN84905610, date: 081214

## Background

The pharyngoesophageal segment (PES) is made up of the inferior pharyngeal constrictor, the cricopharyngeus muscle (CPM) and the proximal part of the cervical oesophagus. The upper oesophageal sphincter (UES) is a 2.5–4.5 cm high-pressure zone visualized on manometry between the pharynx and oesophagus. PES refers to anatomy and UES to function, but the terms are synonymous. The CPM is 1–2 cm and it is a key component of the UES because it is the only portion that actively participates in all reflexive relaxation and tightening activities [1]. Cricopharyngeal dysfunction (CPD), characterized by oropharyngeal dysphagia, may be due to incoordination as well as reduction in maximal opening of the UES during transphincteric flow [2, 3].

Radiological assessment of CPD can be challenging [3]. Videomanometry (VM) combining solid state manometry and videofluoroscopy allows direct comparison of pressure readings with dynamic anatomy giving a better appreciation of how these readings are related to the passage of the bolus [4, 5].

The CPM is frequently targeted for intervention in CPD [6]. There are four approaches to the CPM, including: the external technique, which is indicated when a biopsy is needed; the endoscopic approach, which offers the choice of laser or the surgical stapler; bougie or balloon dilatation of the UES and botulinum toxin injection in the CPM endoscopically [7] or percutaneous [8]. In our department we use balloon dilatation and laser myotomy to treat CPD without Zenker diverticulum.

## Aim of the study

This is a randomized and prospective pilot study to compare the effects of balloon catheter dilatation (BD) and laser myotomy (LM) in CPD.

## Methods

We included patients who had dysphagia due to CPD without Zenker diverticulum for more than 3 months and who had not undergone any previous interventions in the PES. They underwent clinical assessment by an otorhinolaryngologist. None of the patients had medical instability, cervical osteophytes, neurological diseases, untreated reflux or hepatitis. All were informed about the benefits and risk of the procedures and signed an informed consent. After the CPD was confirmed by VM, they were randomized to LM or BD. The study was approved by the ethical committee of the University of Lund.

We evaluated the variables pre- and 1 and 6 months post-treatment using VM and the Swedish version of the Sydney Swallowing Questionnaire (SSQ) [9], a reliable and consistent instrument for the assessment of subjective dysphagia symptoms. The SSQ is a self-report inventory with a maximum possible total score of 1,700; it consists of 17 questions yielding a score of 0–100 for each.

Videomanometry was performed in frontal and lateral projection with the patient seated. Videofluoroscopy was done before inserting the manometry catheter, in order to measure the dimensions of the PES. Then a small amount of topic anaesthetic (Xylocain 2%; Astra Zeneca, Södertälje, Sweden) was placed in the nostril. The catheter was introduced through the nose to UES and oesophagus under fluoroscopic guidance in order to reduce patient discomfort. Time for examination was less than 10 min and total fluoroscopy within 100 s, radiation dose 0.3 mSv. All participants were instructed to swallow 10 ml of water-soluble contrast (Barium contrast medium, 240 mg/ml, Nycomed Imaging, Oslo, Norway) three times. Retention and penetration of the contrast as well as 20 variables were analysed by VM (Table 1).

**Table 1:** Videomanometry variables, all of them in sagittal projection except 1–3

	Preop	Post1	Post2	*P* value	P-value treat
Frontal UES diam.by CPM	9.9	10.6	10.0	0.68	0.78
Frontal UES diam.15 mm over CPM	20.5	20.7	18.7	0.49	0.63
Frontal UES diam.15 mm under CPM	12.0	12.1	13.1	0.21	0.03
UES diam.by the CPM	5.6	7.6	8.4	0.008	0.86
UES diam.15 mm over CPM	13.3	15.2	16.7	0.05	0.29
UES diam.15 mm under CPM	9.6	9.9	11.5	0.16	0.39
Maximal hyoid movement 1	10.9	10.2	11.0	0.77	0.32
Maximal hyoid movement 2	12.5	14.5	16.3	0.11	0.28
Maximal hyoid movement 3	16.5	17.5	18.2	0.67	0.20
Maximal laryngeal elevation	21.4	22.7	24.5	0.61	0.36
Resting UES pressure	65.0	54.4	56.0	0.60	0.92
Residual pressure UES relax dry	3.0	1.3	2.5	0.42	0.54
Residual pressure UES relax wet	3.0	1.3	4.6	0.80	0.73
Duration of UES relax dry	0.5	0.5	0.5	0.68	0.85
Duration of UES relax wet	0.7	0.6	0.7	0.50	0.50
UES contraction pressure	280.0	275.0	293.0	0.95	0.60
Intrabolus pressure	49.0	34.0	–	–	–
Pharyngeal pressure	292.0	302.6	184.0	0.13	0.37
Tongue base pressure	261.0	241.0	187.0	0.02	0.63
Oesophagus amplitude	77.0	82.0	83.0	0.53	0.09

Diameter is measured in mm and pressure in mm Hg. P-value = within subjects, P-value treat = difference between treatments. Maximal hyoid movement is hyoid’s elevation (=maximal hyoid movement 1) followed by a ventral movement (=maximal hyoid movement 2). The diagonal line between the resting position and maximal cranioventral movement is the maximal hyoid movement 3.

*CPM* cricopharyngeus muscle, *Preop* pre-operatively, *Post1* 1 month post-operatively, *Post2* 6 months post-operatively, *UES* upper oesophageal sphincter.

The catheter’s diameter was 4.6 mm with four solid-state pressure transducers positioned 2 cm apart (Konigsberg Instruments Inc. Pasadena, CA, USA). The proximal sensors were dorsal oriented to measure 120°, while the two distal transducers were circumferential, allowing 360° measurements. All sensors were radiopaque and easy to identify during fluoroscopy. The sampling frequency was 64 Hz. The analogue signal was converted to a digital signal (Polygraf, SynMed Medicinteknik, Spånga; Sweden). The pressure values were registered in mmHg and referred to atmospheric pressure. The system was calibrated at 0 and 50 mmHg and carried out at 37°C [10].

Follow-up was made in an outpatient clinic 1 and 6 months after treatment.

Flexible oesophagoscopy was conducted in all patients with reflux symptoms and they received proton pump inhibitors during 2 months preoperatively [11]. Myotomy, using CO_2_ laser, was performed under general anaesthesia, according to the technique described by Lawson [12]. We used neither fibrin glue to the incision nor nasogastric feeding tube to avoid interference with the healing process of the surgical field. During the first postoperative 2 days the patients were fed parenterally. On day 3 a liquid and semisolid diet was authorized and the patient discharged. On day 10 normal diet was resumed. Preoperative temperature, C-reactive protein (CRP), erythrocyte sedimentation rate (SR) and leucocytes were taken and the same procedure was performed 4 h after the operation and in the morning on days 2 and 3 after surgery.

Dilatation was performed with a controlled radial expansion balloon with diameter 18–20 mm, during 2.5 min under general anaesthesia. Temperature and blood test including CRP, SR and leucocytes were taken preoperatively and 4 h postoperatively and in the morning on day 2. If these parameters were normal a liquid and semisolid diet was authorized and the patient discharged. On days 5 to 7 normal diet was resumed.

### Statistics

Data were processed with SPSS version 22 for Mac and statistical analysis was made using descriptive statistics and repeated measures ANOVA, p values <0.05(two-tailed) were regarded as significant.

## Results

Ten patients were included in the study, but only eight patients completed. The mean age was 74 years and the age range 67–81 years. Four participants were male and four female. After being randomized four were treated with BD and four with LM. No complications were reported.

The follow-up time was 1 and 6-months postoperative with SSQ, VM and clinical control in the outpatient clinic.

### SSQ

The response rate to SSQ was 100%. Mean SSQ score (Table 2) was pre-operative: 770 (CI 457–1,084) BD: 691, LM: 850, 1 month post-operative: 340 (CI 74–606) BD: 398, LM: 281 and 6 months post-operative: 559 (CI 212–906) BD: 718, LM: 399 indicating a statistical significant improvement (p = 0.003) in self-reported swallowing impairment, but we could not find a significant difference between the different treatments in our cohort (p = 0.72).

**Table 2:** Pre- and post-treatment Sydney Swallowing Questionnaire´s mean total score presented by case and treatment

Case	Treatment	SSQpreop	SSQpost1	SSQpost2
1	BD	1,142	704	960
2	LM	1,131	756	744
3	BD	648	121	968
4	BD	240	78	86
5	BD	734	691	860
6	LM	1,217	235	736
7	LM	305	24	70
8	LM	748	111	48

*BD* balloon dilatation, *LM* laser myotomy, *SSQ* Sydney Swallowing Questionnaire, *preop* pre-operatively, *post1* 1 month post-operatively, *post2* 6 months post-operatively.

Highest pre-operative mean scores (50 or more) were registered in seven questions: difficulty in swallowing solid food (question 5), difficulty in swallowing dry food (question 6), food gets stuck in the throat (question 9), choke with solid food (question 10), swallowing more than once (question 14), dysphagia severity rate (question 16) and quality of life (question 17). Post-operative mean scores decreased in all these questions, with values equal to 45 or less (Table 3).

**Table 3:** Pre- and post-treatment Sydney Swallowing Questionnaire´s mean scores by question

SSQ Score	Preop	Post1	Post2
1. Swallowing difficulty	36	17	40
2. Thin liquids	32	20	31
3. Thick liquids	26	20	34
4. Soft food	38	19	23
5. Hard food	64	18	42
6. Dry food	61	30	45
7. Swallowing saliva	23	12	17
8. Starting a swallow	47	17	34
9. Food stuck in the throat	65	28	45
10. Cough/choke with solids	60	22	40
11. Cough/choke with liquids	35	19	26
12. Time to eat a meal	33	28	30
13. Food/liquid behind nose	25	12	16
14. Swallow more than once	58	26	40
15. Cough/spit during a meal	44	20	28
16. Dysphagia severity rate	60	21	43
17. Quality of life	68	16	38
Total	770	340	559

*SSQ* Sydney Swallowing Questionnaire, *preop* pre-operatively total score, *post1* 1 month post-operatively total score, *post2* 6 months post-operatively total score.

### Videomanometry

**Figure 1:**
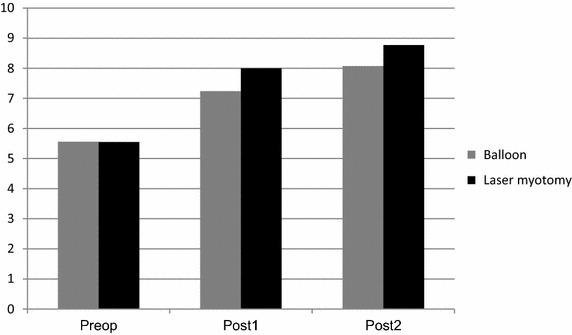
Upper oesophageal sphincter sagittal diameter at cricopharyngeal muscle. *Preop* pre-operatively, *post1* 1 month post-operatively, *post2* 6 months post-operatively.

The UES sagittal diameter at the CPM increased if we considered both treatments, (p = 0.008): pre-operatively mean 5.6 mm (CI 4.1–6.9), BD: 5.6 mm, LM: 5.6 mm, 1 month post-operatively mean 7.6 mm (CI 6.5–8.7), BD: 7.2 mm, LM: 8 mm and 6 months post-operatively mean 8.4 mm (CI 6.4–10.4), BD: 8.1 mm, LM: 8,7 mm, Figure 1, but we could not find a significant difference between the two treatments in our cohort (p = 0.86).

Tongue base pressure decreased (p = 0.02) from pre-operatively: 261 mm Hg (CI 75.3–475) BD: 269 mm Hg, LM: 250 mm Hg, 1 month post-operatively: 241 mm Hg (CI 65.2–432.7) BD: 236 mm Hg, LM: 249 mm Hg, to 6 months post-operatively: 187 (CI 37.7–358.2) BD: 178 mm Hg, LM: 200 mm Hg, without a difference between the treatments in our cohort (p = 0.63).

The pre-operative intrabolus pressure mean value, at the level of the inferior pharyngeal constrictor, was 30 mmHg, because of technical reasons it could not be measured after 6 months in the last four patients and could therefore not be analysed.

Three patients (number 2, 5 and 8) had pre-operative subepiglottic penetration of the contrast in the VM, but none of them had it post-operatively. Three (number 3, 5 and 6) had retention of the contrast in the vallecula pre- and postoperatively. The pre-operative oesophagus amplitude mean was 77 mm Hg, 1-month post-operatively it was 82-mmHg and 6 months post-operatively 83 mmHg.

The following variables did not change post-operatively: Frontal and sagittal diameter of UES, 15 mm over and under the CPM, pharyngeal pressure at the level of the constrictor inferior muscle, maximal hyoid movement, laryngeal elevation, resting UES pressure, residual pressure during UES relaxation dry and wet, duration of UES relaxation dry and wet and UES contraction pressure (Table 1).

## Discussion

Most published studies on CPD treatment are small and retrospective, without randomization and/or control and a short follow-up. Our study is prospective and randomised but with a limited sample size, thus the results should be interpreted with caution.

A postoperative improvement was seen for the SSQ score, UES sagittal diameter at CPM and tongue base pressure after BD and LM. Although we could not find a difference between the treatments in our cohort, patients 1, 3 and 5 treated with BD had high 6 month-post SSQ scores, patients 3 and 5 had increased UES diameter at CPM only 0, 7 mm (Table 4) and these three participants had oropharyngeal dysphagia, made a new VM and required retreatment after 12 months: one with LM and two with new BD. None of the patients who underwent LM have been treated again.

**Table 4:** Pre- and post-treatment UES sagittal diameter at cricopharyngeal muscle

CASE	Treatment	UES preop	UES post1	UES post2	UES post2- UES preop
1	BD	4.7	7.3	7.0	2.3
2	LM	8.3	9.5	10.0	1.7
3	BD	8.3	7.7	9.0	0.7
4	BD	4.7	7.7	11.0	6.3
5	BD	4.6	6.3	5.3	0.7
6	LM	5.3	5.6	6.5	1.2
7	LM	3.3	8.3	6.6	3.3
8	LM	5.3	8.6	12.0	6.7

Measures are made in mm, during bolus passage in videofluoroscopy.

*BD* balloon dilatation, *LM* laser myotomy, *UES* upper oesophageal sphincter, *preop* pre-operatively, *post1* 1 month post-operatively, *post2* 6 months post-operatively.

The success rate of BD varies from 35 to 85% [13]. Considering SSQ and the UES sagittal diameter, the success rate for BD in our study was 100% after 1 month, but only 50% after 6 months.

Balloon catheter dilatation protocols are not yet standardized across institutions [14, 15]. The diameter and pressure of the balloon and the duration of each dilatation varies and appears to be dependent upon the personal preference and the experience of the operator. The UES is kidney shaped which might explain why the dilatation with a cylindrical device only treats part of the sphincter effectively and why it is possible to introduce a 4.6 mm catheter when the sagittal diameter at CPM is only 3.3 mm [16–18, 19]. Cates el al. propose in their study that the circular model underestimates UES area by 60%. The largest dilator currently available for UES dilation is 20 mm. Belafsky et al. in a recent study show how the efficacy of the BD improves using two cylindrical catheters instead of one and they propose a kidney shaped oesophageal dilator [17].

The success rate of myotomy by external approach or by endoscope is around 70% which is in accordance with our results [20]. The ability to recognize the buccopharyngeal fascia, the visceral layer of the middle layer of the deep cervical fascia with the endoscopic technique [21], explains the low rate of complications. We restrict the external approach to cases in which appropriate exposure is impossible to reach via the endoscopic approach.

After an initial compensatory increase of tongue base, intrabolus and pharyngeal pressure at the inferior constrictor, the pharynx becomes progressively dilated and weak proximal to the obstruction as the severity of CPD increases [10]. Frontal and sagittal diameter of UES 15 mm over and under the CPM, pharyngeal pressure at the level of constrictor inferior muscle, maximal hyoid movement, laryngeal elevation, resting UES pressure, residual pressure during UES relaxation dry and wet, duration of UES relaxation dry and wet, UES contraction pressure, did not show any post-operative changes in our cohort (Table 1). These data suggest that once the diagnosis is made, if the comorbidity and functional status of the patient allows the intervention, it should be done before the pharyngeal weakness is irreversible. The length of time over which this may occur is still unknown and is in an area of continuing research [22].

In order to analyse pre- and post-operative changes in UES, we should measure cross sectional dimensions as well, which is not feasible by VM. High-resolution manometry combining oesophageal and pharyngeal impedance and ph-monitoring improves the diagnosis accuracy in the PES and future studies should use these techniques.

## Conclusion

According to measures with both VM and SSQ, LM improves UES opening in 100% and BD in 50% of the patients in our study during at least 6 months. Earlier CPD treatment might relieve symptoms before pharyngeal dimensions change and help to prevent irreversible pharyngeal dilatation and weakness. The success of the procedure is strongly related to the selection of patients.

## Abbreviations

BD: balloon catheter dilatation
CPD: cricopharyngeal dysfunction
CI: confidence interval
CPM: cricopharyngeus muscle
CRP: C-reactive protein
SR: erythrocyte sedimentation rate
LM: laser myotomy
PES: pharyngoesophageal segment
UES: upper oesophageal sphincter
VM: videomanometry
